# Assisted dying in Swedish healthcare: a qualitative analysis of physicians’ reasoning about physician-assisted suicide

**DOI:** 10.1007/s40592-024-00202-5

**Published:** 2024-07-26

**Authors:** Anna Lindblad, Niklas Juth, Ingemar Engström, Mikael Sandlund, Niels Lynøe

**Affiliations:** Karolinska Institute: Karolinska Institutet, Stockholm, Sweden

**Keywords:** Autonomy principle, Physician assisted suicide, Physician attitude, Prescribing lethal drugs, Slippery-slope arguments, Dysphemism, *Primum non nocere*

## Abstract

To explore Swedish physicians’ arguments and values for and against physician-assisted suicide (PAS) extracted from the free-text comments in a postal survey. A random selection of approximately 240 physicians from each of the following specialties: general practice, geriatrics, internal medicine, oncology, surgery and psychiatry. All 123 palliative care physicians in Sweden. A qualitative content analysis of free-text comments in a postal questionnaire commissioned by the Swedish Medical Society in collaboration with the Karolinska Institute in Stockholm. The total response rate was 59.2%. Of the 933 respondents, 1107 comments were provided. The free-text comments entailed both normative and factual arguments for and against PAS. The analysis resulted in two main categories: (1) “Safe implementation of PAS is unachievable” (with subcategories “Criteria of PAS difficult to fulfil” and “PAS puts societal norms and values at risk”) and (2) “The role of PAS in healthcare” (with subcategories “No medical need for PAS”, “PAS is not a task for physicians”, “No ethical difference to other end-of-life decisions” and “PAS is in the patient’s best interest”). The respondents brought up well-known arguments from academic and public debate on the subject. Comments from physicians against PAS were more often emotionally charged and used devices like dysphemisms and slippery-slope arguments.

## Introduction

In Sweden, the legal status of physician-assisted suicide (PAS) is unclear. In an ongoing case, which took place some months before the present study was conducted, a physician who had performed PAS was not prosecuted. The Health and Social Care Inspectorate (IVO) urged, however, for a withdrawal of the physician’s license to practice. At the time of writing, the physician lost his case regarding license withdrawal in three instances, but has now appealed to the supreme administrative court (Bergström 2023).

As in many other countries, PAS has been much debated in Sweden over the years, not least within the medical profession. A diversity of beliefs and attitudes have been expressed, and debaters have offered general ethical arguments as well as arguments more closely related to the role of the medical profession itself (The Swedish Council on Medical Ethics 2017). However, physicians’ attitudes towards PAS have not been scientifically investigated in Sweden since 2007 (Lindblad et al. 2008). Therefore, in 2020, a follow-up questionnaire study was conducted through a collaboration between the Swedish Society of Medicine and Karolinska Institutet.

The quantitative results, which have been presented elsewhere (Lynøe et al. 2021), showed a clear trend towards more accepting views of PAS: in 2020, 47% were in favour of PAS, compared to 35% in 2007 (Lindblad et al. 2008; Lynøe et al. 2021).

The results of the main study were discussed in terms of secular changes in society, differences with respect to the respondents’ ages, and variations among physicians from different medical specialties. A closer look at the free-text comments provided by many of the respondents may shed further light on the underlying values and arguments. In this paper, we present a qualitative content analysis of those comments.

### Aim

To explore Swedish physicians’ arguments and values for and against PAS extracted from free-text comments in a postal survey.

### Method

Manifest qualitative content analysis of free-text comments provided by respondents in a questionnaire on physicians’ attitudes towards PAS (Graneheim and Lundman 2004; Hsieh and Shannon 2005).

### Setting

Postal questionnaires were distributed to randomly sampled physicians from six specialties (240 each from general practitioners, geriatricians, internists, oncologists, psychiatrists, and surgeons) and all registered palliative care specialists in Sweden (*N* = 123), making a total of 1563 physicians of which 933 responded giving a response rate of 59.7%.

The questionnaire contained three questions about the participants’ general attitudes towards PAS: whether they would like to be offered PAS themselves, whether they would be prepared to prescribe the needed drugs to a patient who fulfilled seven specific criteria, and whether they would ask for PAS for themselves, if the same criteria were fulfilled. Response options were Yes, No or Undecided. After each question, space was provided for free-text comments. Respondents were also provided with a set of fixed arguments *pro et con* PAS, but were also encouraged to formulate their own arguments—pro et cons. Finally, at the end of the questionnaire, the participants were invited to give general comments regarding the issue of PAS.

### Data analysis

In total, 1107 comments were provided by a total of 933 unique respondents. Sometimes the comments consisted of arguments, and these have been analysed using qualitative manifest content analysis (Graneheim and Lundman 2004; Hsieh and Shannon 2005). The quantitative results and responses to the fixed arguments have been presented elsewhere (Lynøe et al. 2021, 2022; Lynøe et al. 2021).

Textual data were analysed with descriptive qualitative content analysis using the terminology suggested by Graneheim and Lundman (Graneheim and Lundman 2004) and Hsieh and Shannon (Hsieh and Shannon 2005). Due to the explorative nature of the study, an inductive approach without pre-set categories was chosen. Author NL read all comments (*n* = 1107) separately, abbreviating them into meaning units when needed, and conducted a first coding and categorization in collaboration with AL. After this step, all authors read or re-read the answers, resulting in further development of categories and themes. All authors commented on the analysis, and a discussion followed until consensus was reached.

### Ethical considerations

The project was reviewed and approved by the Swedish Ethical Review Authority, Dnr: 2020-01842.

## Results

The free-text comments entailed normative and factual arguments for and against PAS. The analysis resulted in two categories and six subcategories (Table 1).

**Table 1:**
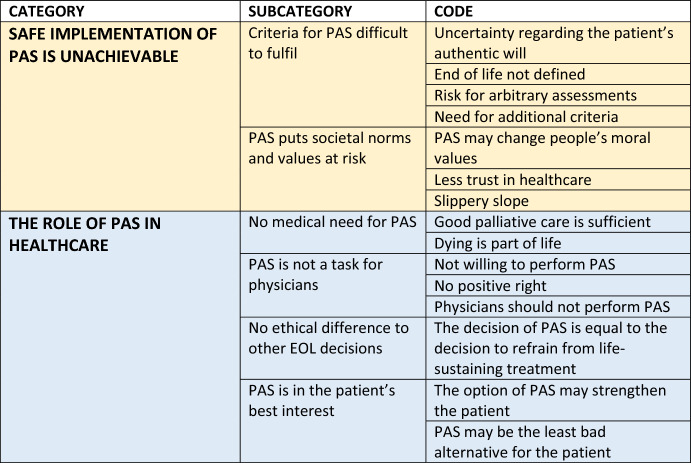
The two main categories are *Safe implementation of PAS is unachievable* (light yellow) and *The role of PAS in healthcare* (light blue)

Subcategories and codes/examples resulting from the manifest content analysis are also listed

### Safe implementation of PAS is unachievable

The first category describes challenges associated with legalization of PAS and contains two subcategories (*Criteria of PAS difficult to fulfil* and *PAS puts societal norms and values at risk*)*.*

#### Criteria of PAS difficult to fulfil

Seven criteria making a patient eligible for PAS were presented in the questionnaire (Appendix 1). These criteria were questioned in various ways in the comments. Several participants brought up the problem of ensuring that the request for PAS is expressing the authentic will of the patient. There were also worries about the risk of pressure from relatives or from society at large, as well as other patient factors that could influence decision competence negatively, such as depression or pain.

“Hard to judge whether a decision has been affected – decision competence and mental state hard to evaluate.” (743)

It was also claimed that it may be difficult to predict precisely when a patient will die, and that ‘end of life’ is a time frame lacking a clear definition. Physicians commented that there was a risk for arbitrary decision-making due to the complexity in assessing the criteria for PAS. There were also concerns that in the case of incorrect assessment of criteria, a patient would wrongly be considered eligible, which could not be undone.

#### PAS puts societal norms and values at risk

This subcategory includes arguments regarding potential negative effects of PAS on individuals or society if PAS should come to be legalized. “*Values may change” (175)*, it was claimed; “*physicians may lose their moral compass” (105)* and the “*elderly begin to conceive themselves a burden to society” (*Lindblad et al. 2008). Furthermore, “the *public’s trust in healthcare may decrease” (224)*.

“Patients would no longer feel safe knowing that healthcare always intends to help them to feel better/relieve/support and not to harm. They would have to consider that the physician also is legally permitted to prescribe drugs to end peoples’ lives.” (670)

Some participants raised concerns that the legalization of PAS could have negative consequences for physicians. Physicians participating in PAS might get used to the practice and thereby become more cynical, not only in relation to patients asking for PAS, but also more generally towards other patients. In the long run, legalizing PAS could therefore have negative consequences for trust in physicians and in the healthcare system as a whole.

The negative effects of PAS, it was argued, could also lead to what is commonly known as the slippery slope (Helgesson et al. 2009), i.e., when the first step in a certain direction is taken, further steps in an unwanted direction are inevitable. Criteria for PAS could be broadened, and legalization of PAS could be followed by legalization of euthanasia.

“One treads on a slippery slope – indications are broadened.” (191)

### The role of PAS in healthcare

The second category concerns PAS’ place in healthcare and contains four subcategories (*No medical need for PAS*, *PAS is not a task for physicians*, *No difference to other end-of-life decisions* and *PAS in the patient’s best interest*). The category includes reasoning for and against PAS as a medical intervention; why, when, for whom or by whom.

#### No medical need for PAS

This subcategory includes arguments that are primarily based on the view that *“death and dying is a natural part of life” (387, 714).*

The theme also includes arguments saying that good palliative care is a sufficient option in healthcare at the end of life, making PAS superfluous. It is claimed that palliative care in Sweden is of such high quality that there is no medical need for PAS, and like other optional medical treatments, PAS cannot be required by patients.

“There is good palliative care, as well as sedation.” (10)

Some respondents with experience of palliative care stated that patients in a palliative care unit sometimes request PAS or continuous deep sedation, but after effective treatment and relief from suffering (e.g., pain), the wish to die will disappear.

#### Not a task for physicians

“We shall cure, relieve and comfort and we shall not prescribe such drugs.”* (296)*

Several respondents stated that PAS is an action that physicians should not be involved in. Several respondents said that they would feel like “murderers” (318, 737) if they participated in PAS.

“This is nothing for the medical services - lawyers should administer and soldiers effectuate.” (357)

Some respondents stated that since there is no legal way to conscientiously object in Sweden, they might be forced to participate in PAS if it were legalized, and if so, they would give up their careers as physicians.

A common reference was the Hippocratic Oath and the axiom “first of all do not harm” (*primum non nocere*); moreover, it was claimed that the Hippocratic Oath was “*crystal clear*” (189) regarding the matter of PAS. It was also pointed out that a legislation of PAS might create situations in which patients feel they are entitled to have PAS or that it might become a duty to request PAS.

“Difficult to exclude outer or inner pressure – feeling as a burden for relatives or believe that relatives might find it better if I die.” (825)

Some respondents also stated that a patient who wanted to commit suicide due to unbearable suffering at the end of life could do so without involving physicians. Others commented on the current societal focus on suicide prevention. Several respondents found it difficult to combine the aim of eliminating suicide with a legalization of PAS.

“Both prevent suicide and assist somatically ill patients to commit suicide?” (12)

#### No ethical difference to other end-of-life decisions

Whereas many physicians raised concerns about PAS, some claimed that assisted dying implemented in the way described in the questionnaire was morally no different from other procedures at the end of life.

”Would prescribe drugs in same way as I respect a patients’ wish to end haemodialysis.” (386)

Since withdrawing life-sustaining treatment is legal in Sweden and can even be done by a patient’s own request, it was considered logical that PAS should also be legalized; both measures have the same outcome in terms of the death of the patient. Moreover, some participants asked how it was possible to harm a patient who was suffering unbearably when refractory symptoms were being treated by offering the patient PAS.

#### PAS is in the patient’s best interest

A palliative care physician in favour of PAS highlighted the need for a humble and person-centred approach by asking:

“How do we assess another human being’s suffering/needs?” (71)

Several participants also stated that legalizing PAS might result in empowering patients and increasing their control over their own end-of-life process. A patient might take the prescribed drugs but could also choose to abstain.

Other participants stated that PAS often is seen as the ‘least bad’ alternative for a patient and that reducing the length of time treating refractory symptoms through PAS should not be considered as harming the patient.

“Letting the patient suffer is ignoring the principle of non-maleficence. Animals are not treated that way.” (471)

Several respondents commented that it is not possible to harm a suffering patient at the end of life by offering PAS and commented on the non-maleficence principle in this context:

“Seen from the patient’s perspective such a patient is not harmed.” (313)

#### Dysphemistic language

Several comments included value-laden negative language and words like ‘murder’, all of which had been written by respondents who were against PAS. Three types of dysphemisms could be identified (Table 2).

**Table 2:** Value-laden terms used in 19 out of 1107 comments (1.9%)

Dysphemistic names for PAS	Dysphemistic metaphors for PAS	Dysphemistic names for the physician
Execution	Mobile gestapo teams	Murderer
Murder	Encounter a murderer	Executioner
Homicide	Playing God	
Manslaughter		
Killing		
Criminal act		
Taking life		

The terms have been categorized into three groups: naming the action itself, metaphors for PAS, and naming the physician

For some of the expressions used, it was possible to infer an underlying religious motive for the dysphemism. Some respondents referred to the Christian Bible and the fifth commandment (141, 190), whereas another stated that because he/she considered him/herself to be a Christian, participating in the killing of a fellow human being was not an option (95). One respondent referred to the sanctity of life (190), and another said that death is a natural part of life, and that physicians should not intervene in the dying process, otherwise it might be understood as playing God (674).

## Discussion

The content analysis revealed arguments that are well known from the public debate about PAS, and similar to the results from a previous study conducted in 2007 (Helgesson et al. 2009). However, the analysis also identified value-based tensions that deserve to be discussed more closely. It is also relevant to relate the content in some of the stated arguments to empirical facts and experiences from healthcare in Sweden.

### No medical need for PAS?

Several respondents argued that there is no medical need for PAS, since good-quality palliative care, including continuous palliative sedation (CDS), is sufficient to meet the needs of patients at the end of life. This is a well-known claim from the debate on assisted dying. According to a recent study, approximately eight percent of patients in specialized palliative care in Sweden receive palliative sedation at the end of life (Hedman et al. 2022). Nevertheless, data from the Swedish quality register on palliative care reveals that many patients in Swedish healthcare (all settings) have exhibited severe symptoms of suffering in the last week of life: severe pain has been registered in about 25% of cases and anxiety in about 13%; severe nausea or dyspnoea are less frequent but occur (The palliative register (in Swedish - Palliativ registret) 2019). So even if good quality palliative care may suffice in most cases, the access to such care is still limited, and sedation at the end of life less common in Sweden than in other countries (Kagan 2012; Oregon Death with Dignit 2020).

The discrepancies found between the prevalence of severe symptoms of suffering in the end of life and the forms of palliation given point to a possible unfairness in the health care system. Some palliative care physicians might be generous with interventions lika CDS or PAS whereas others may be more restrictive under the same medical conditions. Thus, the care given at the end of life might become arbitrary and depending on which palliative care physician the patient happens to meet (Lynøe 2014).

Also, the question remains whether palliative care can meet all needs for all patients. According to data from Oregon, more than 90% of all patients receiving PAS have previously been offered palliative care, suggesting that there might be situations where such care is not enough (Oregon Death with Dignit 2020). Nevertheless, the question remains whether treatment of refractory suffering at the end of life is to be solved by assisted dying.

### Not a task for physicians?

In the Swedish healthcare setting, there is no legal possibility for healthcare professionals to conscientiously object to medical procedures (Lynøe 2014). For that reason, some of those against PAS said that legalization of PAS would force them to decide to quit as clinicians. Our previous study indicates that a significant proportion of physicians would be prepared to participate in PAS were it possible to conscientiously object (Lynøe et al. 2021). Also, the results are in line with a previous study, where physicians argued that PAS would not fit into their professional role (Helgesson et al. 2009).

When arguing against PAS as a physician’s task, several comments referred to medical ethics, some being more specific in mentioning “good” medical ethics and the Hippocratic Oath—the latter probably derived from the Pythagorean tradition, suggesting that physicians should abstain from: (1) using knives, (2) prescribe poisons and (3) abstain from performing abortions (Edelweis 1943). One participant claimed that the Hippocratic Oath is “crystal clear”, referring to the maxim “first of all do no harm” (*primum non nocere*).

So, according to the Hippocratic Oath, a physician should help their patients and avoid harming them. However, to avoid harm should not necessarily be understood as to avoid death at all costs, since a life with unbearable suffering that cannot be sufficiently alleviated could arguably be claimed to be worse than death (Kagan 2012).

### The fifth commandment

Several respondents referred in their answers to the fifth commandment and also mentioned that they were Christians. This leads to the question about the influence of personal convictions and faith in their answers in the questionnaire. In a study from 2010, UK medical practitioners were asked about their religious beliefs and (among other things) their inclination to perform CDS (Seale 2010). Here, those who considered themselves to be non-religious were significantly more inclined to offer patients CDS than those who considered themselves very religious (Seale 2010). It is probable that also Swedish physicians who perceive themselves as religious might let such values influence their medical decision-making. Other studies about Swedish physicians indicate that non-official (that is, personal) values might influence clinical decision-making ‘through the backdoor’ by colouring the physician’s perception of the relevant factual claims and/or observations and assessment of the patient’s suffering or competence (Lynøe 2014).

### PAS and suicide prevention

Some respondents claimed that it would be difficult for physicians to combine PAS with some other medical tasks, such as the prevention of suicide. This argument can be seen in the light of the so called “vision Zero for suicide”, which was adopted by the Swedish parliament in 2008. According to the Vision Zero, ““No person should find him- or herself in a situation in which they experience that the only solution is suicide. The government’s goal is also that no person should take his or her own life” (Socialdepartementet. Prop. 2007). Although much debated, the Vision Zero has influenced healthcare professionals, especially within psychiatry (Karlsson et al. 2018). That some may perceive PAS and suicide prevention as incompatible entities may therefore be understandable.

### Risks associated with the practice of PAS

Many arguments against PAS focused on risks associated with the practice, both regarding the clinical situation (for instance the assessment of decision-capacity and authenticity) and wider perspectives (including detrimental societal changes and reduced trust in healthcare).

Concerns about the problem of authenticity often took the form of descriptions of hypothetical situations in which patients arriving at specialized palliative care units would initially demand PAS, but after adequate symptom treatment, withdraw this request. Hence, legislations allowing PAS usually demand that the patient’s request for PAS is stable over time, and that other treatment options have been considered (Rogmark and Lynøe 2021).

Several comments seen in this study invoked slippery-slope arguments (asking, for example, what would happen in the long run if PAS was legalized). Such arguments are usually constructed so that the first step is not necessarily bad, but the consequences of the next or next-next step are undesirable or catastrophic (Helgesson et al. 2017). However, the format of this study makes it difficult to interpret why the respondents promoted these undesirable scenarios. Earlier studies give reasons to believe that these prognostications may be coloured by the physician’s own private values and/or emotional reactions (Helgesson et al. 2017): some of these respondents may have disliked PAS from the beginning, and therefore used conjectures about what could happen in the future if PAS was legalized to underpin their view that PAS is bad (Helgesson et al. 2017).

### The patient’s interests

Several participants stressed that respecting a patient’s right to participate in decision-making is important and that PAS could be understood as empowering such a right. That assisted dying is a question of self-determination is a common argument among those in favour of PAS, and one could claim that this argument is in line with a general trend of greater focus on the principle of autonomy (Gillon 2003). In Sweden, this has been embodied in the Patient Law, which regulates the rights of patients to shared decision-making in healthcare (Patientlag 2014).

Some participants also questioned whether providing PAS upon a patient’s request in a situation of severe suffering actually constituted harm. This line of argument relates to the morally difficult area of what is to count as good and bad in a human life and which is more important, the quality of life or the length of life. It could also be interpreted as a consequentialist approach to the question of assisted dying, thus colliding with the current ethical rules of the Swedish Medical Association, which state that a physician should never take measures where the *intention* is to shorten life (Läkarförbundets etiska regler - Sveriges läkarförbund (slf.se) 2019).

These arguments rest upon the assumption that the person most suited to decide about what measures to take is the patient in question. At present, Swedish public debate about patient rights at the end of life is often limited to PAS. However, one might argue that putting patient interests first also implies broadening the discussion to involve questions about CDS and other palliative sedation strategies.

### The physician’s interests

Respondents not only described PAS as a measure that could be in a patient’s best interest, but also as a measure that could be in a physician’s interest. For instance, several participants stated that PAS could sometimes be the least bad option, indicating that they perceived a dilemma between not being able to offer an unbearably-suffering patient sufficient symptom-relief on the one hand, and offering PAS on the other.

One respondent commented that it was important to protect a patient from suffering and at the same time protect that patient’s autonomy. This claim raises a question: is it more important to *protect* and *preserve* a patient’s autonomy, or to *respect* it in accordance with the autonomy principle? If the latter is considered more important, the consequences might be that such a patient cannot be offered any treatment that reduces autonomy, thus excluding any form of palliative sedation as well as PAS. It has been argued that this line of argument might be considered similar to paternalism in the name of autonomy (Lynøe et al. 2021).

### Strengths and limitations

That the materials were collected from free-text spaces in postal questionnaires implies both strengths and limitations. Some comments were short and there is a risk of misunderstanding them. In contrast to a personal qualitative interview, there is no opportunity to pose follow-up questions and ask for clarifications and examples. On the other hand, comments on an anonymized postal questionnaire are often written without filters and without the respondent trying to figure out what answers the interviewer wants to hear. But the question is whether the pre-structured part of the questionnaire might have influenced or even provoked some responders and thus influenced their comments. The questionnaire included fully formulated arguments pro and con PAS for the respondents to agree or disagree with. This might to varying extent have influenced the content of their comments. In some instances dysphemisms were used, which might be a result of a respondent’s feeling that ticking the do not agree-box was not enough to express his or her distancing.

## Conclusions

The physicians in this study brought up the most well-known arguments from both the academic and the public debate regarding assisted dying. Arguments for PAS included for instance that there is no ethically relevant difference between PAS and ending life-sustaining treatment, or acting in the best interest of the patient with regard to his/her autonomy and quality of life. Arguments against PAS highlighted risks, such as the criteria for PAS being difficult to fulfil, that PAS puts societal norms and values at risk, that there is no medical need for PAS, or that assisting suicide is not a task for physicians. Physicians against PAS provided many more comments, as well as more emotionally charged comments, for instance using dysphemisms.
